# BDNF Val^66^Met Polymorphism Influences Visuomotor Associative Learning and the Sensitivity to Action Observation

**DOI:** 10.1038/srep34907

**Published:** 2016-10-05

**Authors:** Vincent Taschereau-Dumouchel, Sébastien Hétu, Pierre-Emmanuel Michon, Etienne Vachon-Presseau, Elsa Massicotte, Louis De Beaumont, Shirley Fecteau, Judes Poirier, Catherine Mercier, Yvon C. Chagnon, Philip L. Jackson

**Affiliations:** 1 École de psychologie, Université Laval, Québec, G1V 0A6, Canada; 2 Centre interdisciplinaire de recherche en réadaptation et intégration sociale (CIRRIS), Québec, G1M 2S8, Canada; 3 Centre de recherche de l’institut universitaire en santé mentale de Québec (CRIUSMQ), Québec, G1J 2G3, Canada; 4 Human Neuroimaging laboratory, Virginia Tech Carilion Research Institute, Roanoke, VA, 24016, USA; 5 Department of physiology, Northwestern University, Chicago, 60611, I1. USA; 6 Departement de psychologie, Université du Québec à Trois-Rivières, Trois-Rivières, Québec, G9A 5H7, Canada; 7 Centre de recherche de l’Hopital Sacré-Coeur, Montréal, Québec, H4J 1C5, Canada; 8 Department de réadaptation, Université Laval, Québec, G1V 0A6, Canada; 9 Berenson-Allen Center for Noninvasive Brain Stimulation, Beth Israel Deaconess Medical Center, Harvard Medical School, 330 Brookline Ave, Boston, MA, 02215, US; 10 Department of psychiatry and medicine, McGill University, Montréal, Québec, H3A 1A1, Canada; 11 Douglas Mental Health University Institute, Verdun, Québec, H4H 1R3, Canada; 12 Département de Psychiatrie et des Neurosciences, Université Laval, Québec, G1V 0A6, Canada

## Abstract

Motor representations in the human mirror neuron system are tuned to respond to specific observed actions. This ability is widely believed to be influenced by genetic factors, but no study has reported a genetic variant affecting this system so far. One possibility is that genetic variants might interact with visuomotor associative learning to configure the system to respond to novel observed actions. In this perspective, we conducted a candidate gene study on the Brain-derived neurotrophic factor (BDNF) Val66Met polymorphism, a genetic variant linked to motor learning in regions of the mirror neuron system, and tested the effect of this polymorphism on motor facilitation and visuomotor associative learning. In a single-pulse TMS study carried on 16 Met (Val/Met and Met/Met) and 16 Val/Val participants selected from a large pool of healthy volunteers, Met participants showed significantly less muscle-specific corticospinal sensitivity during action observation, as well as reduced visuomotor associative learning, compared to Val homozygotes. These results are the first evidence of a genetic variant tuning sensitivity to action observation and bring to light the importance of considering the intricate relation between genetics and associative learning in order to further understand the origin and function of the human mirror neuron system.

More than two decades ago, the discovery of neurons in the macaque brain that respond both to action execution and to action observation sparked a revolution in social neuroscience^1^,^2^. These neurons have since been shown to be involved in the transformation of visual information into fine-grained changes in the motor system (see ref. 3). In humans, this fine-grained tuning is evidenced by the fact that observing a hand movement will not result in a general increase in excitability of all hand muscle representations within the primary motor cortex (M1) but only of those involved in the observed movement (for a review, see ref. 4). From a signal detection theory standpoint^5^, this property suggests that the human mirror neuron system (MNs) is somehow tuned to exhibit sensitivity to observed actions (i.e., muscle-specific motor facilitation induced by action observation). Here, the sensitivity to observed actions is used as an indirect measure of the activity of the MNs in line with previous findings that linked muscle-specific motor facilitation to the human premotor and parietal regions where mirror responses have been commonly reported^6^,^7^,^8^,^9^,^10^.

Despite sustained scientific efforts, many questions remain regarding the origin of this system and its tuning. Many believes that coarse connections present at birth between visual and motor neurons are refined during development by contiguous and contingent activation of visual and motor neurons so that motor neurons become “tuned” to specific observed actions and not to others^11^. This associative learning process has been shown in adults by important work demonstrating that visuomotor associative learning can reconfigure the motor facilitation evoked by action observation (see ref. 12). Interestingly, recent work by Ubaldi and colleagues^13^ and by Barchiesi & Cattaneo^14^ indicated that, in human adults, associative learning can reconfigure the later motor response occurring around 300 ms while the earlier motor response occurring around 150 ms might remain unchanged by associative learning paradigms.

Associative learning has received considerable support in recent years, but many have proposed that other mechanisms might also intervene to canalize and stabilize the development of the MNs (i.e., to allow motor representations to develop sensitivity to corresponding observed actions and to stabilize this sensitivity over time)^15^,^16^. For instance, the developmental canalization hypothesis^15^ and the epigenetic hypothesis^16^ propose that the MNs would rely on associative learning early in life, but that genetic and epigenetic factors would also canalize and stabilize the tuning of the MNs so that motor representations would remain particularly sensitive to corresponding observed actions. This perspective is akin to the notion of critical developmental windows that allows, for instance, the neurons in the primary visual cortex to acquire a specific tuning that will later remain mainly unchanged outside of this time window^17^. Interestingly, this view has also received recent support from the literature on multisensory integration. Accordingly, while many studies have shown that interactions between the senses are quite plastic during childhood^18^,^19^,^20^, recent data suggests that the multisensory binding tendency stabilizes in adulthood^21^. This suggests that multisensory binding tendencies should remain mainly unchanged in adulthood even if learning can transiently alter them.

Interestingly, while genetic^12^,^15^,^16^ and epigenetic^16^ factors have been extensively discussed theoretically in the previous literature, no study has yet reported an actual association between a specific genetic variant and the MNs. The fact that there are important individual differences in the sensitivity (muscle-specific cortico-spinal facilitation) to action observation in human (e.g. ref. 22,23) suggests that genetic variants may indeed play a role in the tuning of this system. Much like the development of orientation selectivity in the visual system^24^,^25^,^26^, learning may interact with specific neurobiological networks to allow motor representations to develop sensitivity to specific observed actions. Accordingly, a genetic variant affecting the neurobiology of learning in the MNs may affect this tuning. A likely candidate is the brain-derived neurotrophic factor (*BDNF*) Val66Met polymorphism (196 A/G; rs6265), a single nucleotide polymorphism frequently associated with a shift from plasticity to stability in neural circuits^27^ and notably, to experience-dependent plasticity in M1, the cortical region mainly targeted in TMS studies of action observation in human^28^,^29^. Importantly, this functional polymorphism affects activity-dependent secretion of BDNF^27^,^30^ and could therefore play a major part in the tuning of the MNs by interacting with associative learning.

In a single-pulse TMS study, we investigated the role of the *BDNF* Val66Met polymorphism (1) on the sensitivity to action observation and (2) on associative learning, evidenced by changes in sensitivity following visuomotor associative learning. In a double-blind procedure (i.e., neither the participants nor the experimenters were aware of genotypes), the participants’ baseline response to action observation was first assessed in a TMS session. Then participants completed a visuomotor associative learning session aimed at reconfiguring the tuning of sensitivity. Finally, a second TMS session was carried out to measure the effect of the learning session on action observation (see Fig. 1). We hypothesized that Met participants would exhibit less sensitivity to action observation during the pre-training session. Also, after assessing the behavioural and neurophysiological markers of associative learning (i.e., response time change and sensitivity change) across genetic groups, we measured the effect of genotype on these markers and hypothesized that Met participants would present less associative learning than Val/Val participants. We aimed at studying the effect of the *BDNF* Val66Met polymorphism on “late” motor facilitation (320 or 640 ms) since associative learning as been shown to affect motor facilitation primarily in this time window^6^,^14^,^31^,^32^,^33^ while having no effect on “early” motor facilitation^13^,^14^.

## Results

### The effect of genotype on sensitivity to action observation

From a signal detection theory perspective, in order for motor facilitation to be considered muscle-specific it should be sensitive enough to distinguish, solely on the basis of its activity, between two action observation conditions. In fact, a good tuning of the system would maximize the sensitivity of motor representations to discriminate between the observation of a compatible action (signal) and an incompatible action (noise) (see Data analysis). In order to measure the sensitivity of the FDI and ADM representations, we opted for the sensitivity index (d-prime)^5^, the most common measure of sensitivity in signal detection theory (see Figure S1 [Supplementary Material] for group averaged Motor-evoked Potentials (MEPs)). To determine the sensitivity of the MNs to discriminate between compatible and incompatible conditions within-participants, we averaged the sensitivity index (d-prime) of pre-training blocks and tested the effect of genotype on this measure. The results of a mixed-design ANOVA with Genotype (Val/Val; Met) as a between-subject factor and Muscles (FDI; ADM) as a within-subject factor indicated a main effect of genotype (F(1,30) = 4.32, *P* = 0.046; partial η^2^ = 0.13) with Val/Val participants presenting significantly greater d-primes (*M* = 1.18; *SD* = 0.*14*) than Met participants (*M* = 1.09; *SD* = 0.*08*) (see Fig. 2). Furthermore, the results indicated a significant main effect of Muscle (F(1,30) = 4.69, *P* = 0.038; partial η^2^ = 0.14) suggesting that d-primes in the FDI muscle were significantly greater (*M* = 1.17; *SD* = 0.14) than d-primes in the ADM muscle (*M* = 1.10; *SD* = 0.16). No Muscle X Genotype interaction was observed (F(1,30) = 1.57, *P* = 0.22). These results provide evidences for an effect of the *BDNF* Val66Met genotype on the tuning of sensitivity to action observation, as Met participants presented significantly less sensitive motor facilitation than Val participants during action observation.

To ensure that these results were not related to differences in muscle activity prior to TMS pulses, a control analysis was conducted on the root mean squares (RMS) of the EMG signal prior to stimulation (see Procedure). The results of a mixed-design ANOVA with Genotype (Val/Val; Met) as a between-subject factor and Conditions (little finger movement, index finger movement, or no movement) and Muscles (FDI; ADM) as within-subject factors revealed no significant main effect or interaction (all *ps* > 0.10), which indicates no difference in RMSs between muscles, conditions or genotypes. Furthermore, to determine the association of genotype with a non-specific effect of TMS, independent sample t-tests were carried on MEP amplitudes in the baseline condition (receding hand). No between-group difference was observed for the FDI (*t* (30) = 0.93, *P* = 0.93) and the ADM muscles (*t* (30) = 1.27, *P* = 0.22), which indicates an absence of non-specific effect of genotypes on MEPs.

### The effect of associative learning

Associative learning was hypothesized to change response time during the learning task as well as sensitivity after the learning task (see Procedure). We first aimed at establishing the effect of the learning session on these two measures before determining how genotype can influence these markers of associative learning.

First, we tested the effect of associative learning on response time change between the first and last blocks of the associative learning task (Fig. 3a,b). We conducted a repeated-measure ANOVA on response time with two within-subject factors of Time (First and last block of the learning session) and Muscles (FDI; ADM). The results revealed a significant main effect of Time (F(1,28) = 61.29; *P* < 0.001; partial η^2^ = 0.69) with significantly shorter reactions time in the last block (*M* = 539.01 ms; *SD* = 108.37) in comparison to the first block (*M* = 649.97 ms; *SD* = 140.62). The results also revealed a significant main effect of Muscle (F(1,28) = 24.12; *P* < 0.001; partial η^2^ = 0.46) with significantly longer reaction times in the FDI muscle (*M* = 611.23 ms; *SD* = 123.16) as opposed to the ADM muscle (*M* = 577.74 ms; *SD* = 118.80). No Time X Muscle interaction was observed (F(1,28) = 0.396; *P* = 0.53). This decrease in response time between the first and last blocks of the task indicates the presence of motor learning during the associative learning task.

Second, we investigated the effect of associative learning on sensitivity change without considering genotypes. To obtain the most stable estimate of pre-training sensitivity, all pre-training blocks were averaged since there was no difference between the first half and the last half of pre-training blocks (F(1,31) = 0.138; *P* = 0.713), which indicates stability in pre-training sensitivity. We then conducted a repeated-measure ANOVA on d-primes with two within-subject factors of Time (Pre-training; First half of post-training blocks; Second half of post-training blocks) and Muscles (FDI; ADM). The post-training blocks were divided in first and second halves to assess the stability of the effect (see Procedure). The results revealed a significant main effect of Time on d-primes (F(1.88, 56.48) = 3.697; *P* = 0.033; partial η^2^ = 0.15) but no Time X Muscles interaction (F(1.94, 58.33) = 0.670; *P* = 0.51). Since no Time X Muscles interaction was observed, muscles were pooled to study the time effect. Post-hoc comparisons, carried using the Bonferroni correction indicated that the first post-training blocks presented significantly lower d-primes (*M* = 1.05; *SD* = 0.13) than pre-training blocks (*M* = 1.13; *SD* = 0.12) (*t* (30) = 2.743; *P* = 0.01; Cohen’s d = 0.5), indicating a learning effect in the first pre-training blocks. Interestingly, the effect of the associative learning task seemed to be transient as the last blocks of the post-training session were not significantly different from the pre-training blocks (*t* (30) = 0.712; *P* = 0.48), which suggests a quick return to baseline sensitivity (see Fig. 3c).

### The effect of genotype on markers of associative learning

To determine the effect of genotype on the markers of associative learning, we first tested the effect of genotype on motor learning. We conducted a repeated-measure ANOVA on response time with two within-subject factors of Time (First and Last block of the learning session) and Muscles (FDI; ADM) and one between-subject factor of Genotype. The results revealed a significant Genotype X Time interaction (F(1,27) = 4.34; *P* =0.047; partial η^2^ = 0.14). We found that participants in the Met group presented less learning during the associative learning task in terms of change in response times between the first and the last training blocks (*M* = −80.1 ms; *SD* = 64.25) when compared to the Val/Val group (*M* = −137.9 ms; *SD* = 77.3) ((F(1,28) = 4.966, *P* = 0.034; partial η^2^ = 0.15; see Fig. 3a,b). This indicates that the Val66Met polymorphism can influence visuomotor associative learning and more precisely motor performance change during the associative learning task.

As our results indicated that associative learning changed sensitivity to observed actions transiently, we focussed our analysis of the effect of genotype on changes in sensitivity between the pre-training and first-half post training. Accordingly, a mixed-design ANOVA was conducted on mean d-primes with two within-subject factors of Muscles and Time (Pre-training; First-half post-training blocks) and the between-subject factor Genotype. The results indicated a significant Time by Genotype interaction (*F*(1, 29) = 5.487, *P* = 0.026, partial η^2^ = 0.16), but no main effect of Muscles (*F*(1,29) = 2.438; *P* = 0.13) nor three-way interactions (*F*(1,29) = 0.086; *P* = 0.77) (see Fig. 3d). To decompose the Time by Genotype interaction, paired sampled t-tests were carried independently on Val/Val and Met participants using the Bonferroni correction. Val/Val participants presented a significant decrease in d-primes between pre-training and the first-half post-training blocks (*t* (14) = 4.062; *P* = 0.001; Cohen’s d = 0.84), whereas Met participants presented no difference (*t* (15) = 0.451; *P* = 0.658). Taken together, these results indicate that the BDNF Val66Met polymorphism can interact with visuomotor associative learning to fine-tune the sensitivity to action observation as changes in sensitivity was only observed in Val/Val participants (see Fig. 3d).

## Discussion

Our results provide the first data suggesting that the *BDNF* Val66Met polymorphism can affect the tuning of sensitivity to action observation. Specifically, Val/Val participants presented more sensitivity than Met participants (monozygotes or Val/Met) before any training. Furthermore, visuomotor associative learning, a process proposed to be at the origin of the MNs and of its tuning, was significantly lower in Met participants compared to Val/Val participants. These results constitute an important evidence to suggest that the sensitivity to action observation may be influenced by specific genetic factors. Moreover, it identifies visuomotor associative learning as a plausible mechanism of action through which this genetic factor could achieve its influence. Furthermore, since the effect of associative learning appeared transient, this indicates that stable tuning patterns might be predominant even if brief periods of training can transiently alter sensitivity. This observation is coherent with theoretical models advocating stabilization processes in the MNs such as the developmental canalization hypothesis^15^ and the epigenetic hypothesis^16^.

There is still a lively debate regarding the origin of the sensitivity to action observation in the human MNs (e.g. ref. 12,16). Our results constitute the first indirect evidence suggesting an interaction between genetic and environmental factors in the tuning of the system as suggested by^16^. One possibility is that the *BDNF* Val66Met polymorphism could affect the tuning of the MNs early in the development by acting on visuomotor associative learning. Many have proposed that coarse connections between visual and motor representations may be present at birth and refined during development when motor and visual representations are paired during imitation^12^,^16^. This process would allow motor representations to develop sensitivity to specific observed actions. Importantly, BDNF has been shown to affect a similar process in the developing visual system. In fact, BDNF is critical for the maturation of the GABA mediated inhibition associated with the development of visual acuity during the early post-natal critical period of visual plasticity^24^. During this process, visual neurons presenting activity to several orientations sharpen their tuning to selectively discharge during the presentation of specific orientation and not during the presentation of others. Importantly, the sharpening of feature-selectivity has been shown to rely mainly on the maturation of inhibitory synaptic transmission^25^ and recent data indicate that activity-dependent expression of BDNF controls the development of the inhibitory synaptic transmission during the critical period^26^.

Similarly, the Val66Met polymorphism may tune feature-selectivity in the MNs by acting on activity-dependent release of BDNF^27^,^30^. Here, motor representations might be first sensitive to many observed actions (see ref. 11). The development of GABA mediated inhibition regulated by the activity-dependent release of BDNF would sharpen the tuning of motor representations so that they develop sensitivity: responding to a given observed action and not to others. This tuning would most likely occur during development because the *BDNF* Val66Met polymorphism affects activity-dependent release of BDNF and would therefore have to interact with experiences to confer its effect. Our results indeed suggest this possibility as the Val66Met polymorphism was not only linked to differences in general sensitivity to action observation measured in adulthood but also to reduced visuomotor associative learning, which suggests a gene-by-environment interaction. This hypothesis could be verified by studying the dose dependent effect of visuomotor associative learning in Met participants. This could be achieved, for instance, by manipulating the number of trials in the training session and by studying the effect of exposure on the sensitivity to action observation. Also, if BDNF affects sensitivity to action observation by acting on GABA mediated inhibition, studying the interaction between these two networks could bring important insights on the origin of the MNs.

One important finding of the current study is the transient nature of the associative learning effect. One explanation for this is that sleep may be necessary to stabilize the effect of learning as previous studies reported strong effects the day following training^6^,^32^. Another possibility is that associative learning in adults may generate rather transient effect without changing stable patterns of motor facilitation learned earlier in development. Our results indeed showed only a transient decrease of sensitivity in Val/Val participants, which raises the question of the stability of motor facilitation in adults. Interestingly, the action of BDNF in the developing visual system may also provide a new perspective on this question. BDNF has indeed been shown to regulate the initiation and closure of the critical period for visual cortical plasticity during which feature selectivity is developed^17^,^24^,^34^. Importantly, outside of this critical period, feature selectivity cannot be substantially altered. Our results are coherent with the existence of such a critical period in the development of the MNs, which is coherent with the developmental canalization hypothesis and the epigenetic hypothesis. Indeed, associative learning in adults may interfere with motor facilitation and lead to a transient decrease of sensitivity, but may allow a limited development of durable sensitivity to new observed actions. In this view, *BDNF* Val66Met may influence the tuning of the system as well as the opening and closing of the critical developmental window, but may have limited effect to reconfigure the long lasting sensitivity of the system in adults. In fact, previous studies convincingly showed that associative learning can specifically alter motor facilitation^6^,^32^, but the important question of the stability of the effect of associative learning still needs to be addressed. Future studies could be conducted on the extinction of visuomotor associative learning, as well as the role of BDNF and overnight motor consolidation in this process.

It is important to point out that our results do not indicate a causal relation between the *BDNF* Val66Met and the indirect measures of the MNs used in this study. Co-inherited SNPs that are in linkage-disequilibrium with the *BDNF* Val66Met could also be associated with the observed effects. It is therefore important to remain cautious when interpreting these results. Furthermore, while the sample size was in line with those used in previous similar studies^6^,^13^,^14^,^32^, further replication is needed to solidify the validity of these findings. Further considerations should also be given to the timing of the stimulations used to assess the effect of the associative learning task because recent works suggest that an effect of associative learning could be observed ~300 ms after stimuli presentation while earlier stimulation delivered ~250 ms does not seem to show a similar modulation^14^. This point appears important as later modulation (~300 ms) appeared to be more controlled/rule-based and anatomically dissociated than early motor facilitation occurring around 150 ms, which in turn appear more automatic/stimulus-driven and maybe more relevant to the MNs^13^. Therefore, we advocate for more work looking into the timing of the effect of the *BDNF* Val66Met on associative learning, as this effect might be related to some specific processes during action observation per se and not necessarily directly to motor facilitation.

In the last decade, BDNF has been widely studied for its role in neurological and psychiatric disorders and is even considered a potential therapeutic target for many neuropsychiatric disorders^35^. The fact that the Val66Met polymorphism influences the tuning of sensitivity to action observation opens important avenues of research to address psychopathologies such as schizophrenia that were previously associated both to the MNs (see ref. 36) and to the Val66Met polymorphism (see ref. 37). Our results bring to light the first specific genetic variant indirectly associated to the MNs and highlights the necessity to study the genetic and environmental underpinnings of this widely studied network in order to better understand the fascinating neurological foundations of human social cognition.

## Materials and Methods

### Participants

Two experimental groups were selected from a larger sample of university students genotyped for the Val66Met polymorphism (n = 111). Participants were told that they would have to provide a saliva sample and complete a few questionnaires. They were also informed that they might be called back to participate in this study. From this pool, 6 Met/Met participants were identified and agreed to take part in the study. 10 Val/Met and 16 Val/Val participants were randomly selected to complete the Met group and Val group respectively. All participants that completed the procedure were included in the analyses. There was no difference between groups on age, gender, or education level (see Table 1). Participants were right-handed, reported no diagnosis of psychopathologies or neurological conditions and presented no contraindications to single-pulse TMS^38^. Only Caucasians of European ancestry were included to avoid population stratification artefacts and race dissimilarity effects documented in an earlier action observation study^39^. All participants provided written informed consent and were compensated for their participation. The local ethics committees (i.e., Comité d’éthique de la recherche de l’Institut universitaire en santé mentale de Québec and Comité d’éthique de la recherche de l’Institut de Réadaptation en deficience physique de Québec) approved the study and the study was conducted in accordance with the approved guidelines.

### Genotyping procedure

Saliva samples were collected using the Oragene DNA self-collection kit (DNAGenotek). DNA was extracted using the Blood & cell culture DNA maxi kit (Qiagen) and concentration evaluated by fluorescence (Qubit). Some 50 nanograms of DNA was used to amplify the region of the *BDNF* Val66Met polymorphism using a real-time polymerase chained reaction cycler (Ligthcycler 480, Roche) and a TaqMan 5′ nuclease assay kit (Life Technologies). PCR was made using 0.5 uL of 10X PCR MasterMix (Roche), 0.25 uL 40X TaqMan assay, 50 ng DNA in a final volume of 5 uL. PCR run included a denaturing step of 10 min −95 °C followed by 35 PCR cycles (1 min 95 °C, 1 min 55 °C, 1 min 72 °C).

### TMS and Motor Evoked Potentials recording

**Figure 1:**
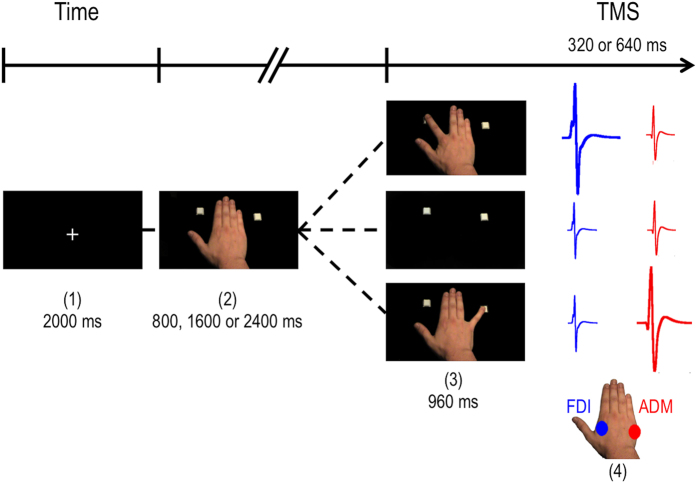
The sequence of a trial of action observation. Each trials consisted of a fixation cross (1) (2000 ms) followed by the dorsal view of a static hand (2) during an interval varying between 800 and 2400 ms. The picture of one of the three experimental conditions (3) was then presented for 960 ms and TMS pulses were delivered at 320 or 640 ms following the onset of the last picture. (4) Schematic representation of sensitivity to action observation (FDI: continuous lines; ADM: dashed lines). Muscle representations are expected to present muscle-specific motor facilitation during the observation of a compatible action (e.g., Index finger observation in the FDI muscle) and not during the observation of an incompatible action (e.g., Little finger observation in the FDI muscle).

**Figure 2:**
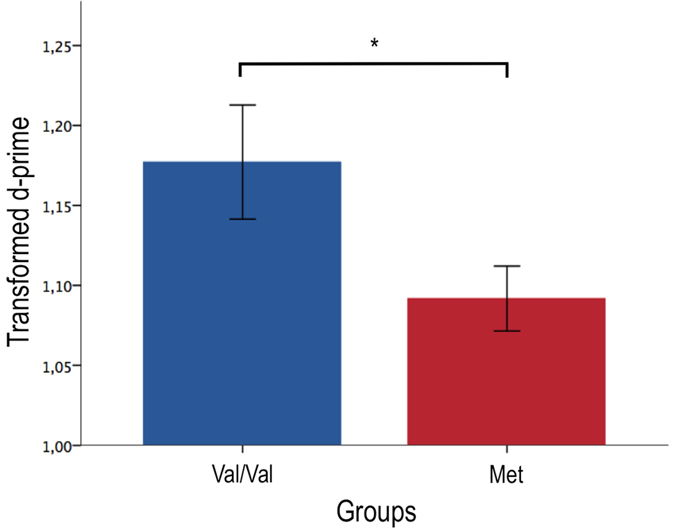
Val66Met polymorphism influences pre-training sensitivity to action observation. Met participants present significantly less sensitivity to action observation before the associative learning session. Error bars indicate standard errors of the mean. Square root transformation were applied to d-prime data for statistical analysis. *P < 0.05 (see also Figure S1 and S2).

**Figure 3:**
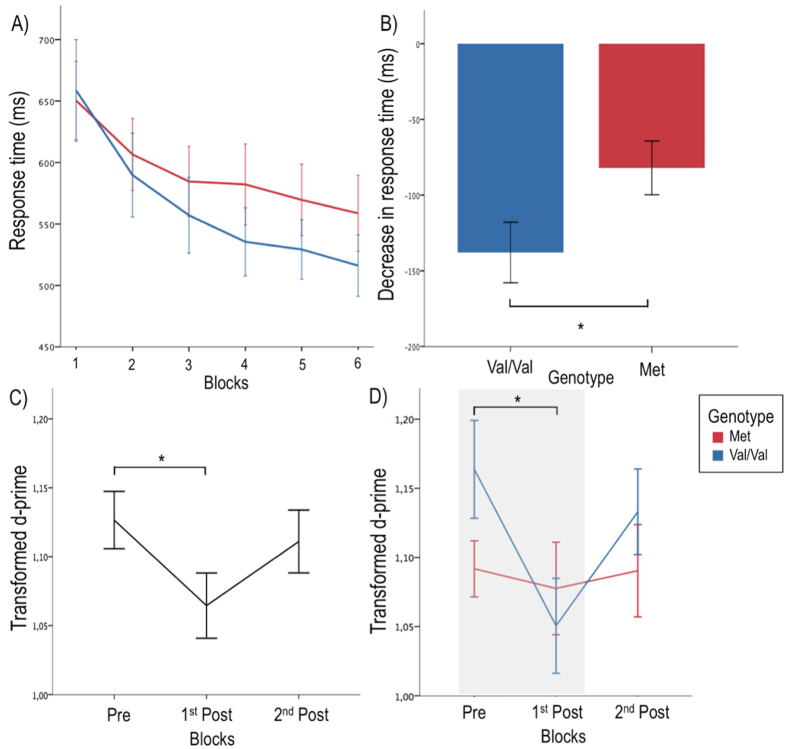
Val66Met polymorphism influences markers of visuomotor associative learning. (**A,B**) Val/Val participants presented a significantly greater decrease in response time between the first and the last blocks of the associative learning task when compared to Met participants (p = 0.034). (**C**) Significant decrease in sensitivity across genetic groups between Pre-training blocks and First half of post-training blocks (*P* = 0.01). (**D**) Significant decrease in sensitivity between the First half of post-training blocks and Pre-training blocks in Val/Val participants (p = 0.001). Met participants presented no decrease in d-prime between the two sessions (p = 0.658), which would indicate visuomotor learning even in the context of weaker pre-training d-primes (see procedure). Error bars represent standard errors of the mean. Square root transformations were applied to d-primes for statistical analysis. *P < 0.05 (see also Figure S3).

MEPs were recorded from the *first dorsal interosseous* (FDI) of the right index finger and the *abductor digiti minimi* (ADM) of the right little finger (see Fig. 1). Monophasic magnetic stimulations were delivered over the left M1 using a 70 mm figure of eight coil connected to a BiStim^2^ stimulator (Magstim Company Ltd., Whitland, UK). Coil orientation was tangential to the scalp with the handle pointing backward and laterally at approximately 45 degree away from the midsagittal line. Coil positioning was guided by the Brainsight^TM^ neuronavigation system (Rogue Research, Montreal, Canada) using a template MRI to ensure accurate coil repositioning between blocks. The coil position and stimulation intensity were determined using a method described by Catmur and colleagues^6^. First, the optimal site was determined by finding the stimulation site where the smallest stimulation intensity could trigger activity in both muscles simultaneously. The intensity of stimulations was then adjusted to produce MEPs of about 1 mV in both muscles during the experiment^40^. MEPs were recorded using surface Ag/AgCl electrodes placed in a belly-tendon montage and a ground electrode on the right wrist. Electromyographic signals were sampled at 2000 Hz, amplified (X1000) and filtered (band-pass 20–1000 Hz) using a NTI amplifier and a 16 bits analog/digital converter (Power 1401, Cambridge Electronic Design, Cambridge, UK). Signals were recorded using homemade functions implemented in the Spike 2 software (Cambridge Electronic Design) and saved for offline analyses.

### Procedure

The experiment was conducted in a double-blind procedure (i.e., neither the participants nor the experimenters were aware of genotypes), in one visit at the laboratory, and divided in three parts: a first TMS session of action observation (pre-learning), an associative learning session and finally, a second TMS session of action observation (post-learning). In the action observation sessions (pre- and post-learning), participants were seated in a dimly lit room with their right hand positioned across their body so that their forearm was parallel to the computer screen to avoid spatial compatibility effects with the visual stimuli^41^. They were instructed to passively observe stimuli in three conditions: 1) little finger abduction; 2) index finger abduction; 3) baseline (see Fig. 1). Participants were asked to pay attention to the finger movement as some trials would contain the presentation of a target (i.e., a circle of ~1^o^ of visual angle) close to the finger and they would have to report it verbally. Each trial started with the presentation of a fixation cross followed by a dorsal view of a right hand for a variable duration (800, 1600 or 2400 ms). The image was then replaced with an image from one of the three experimental conditions for 960 ms, and TMS pulses were delivered at variable intervals (320 or 640 ms) after this image onset to prevent the prediction of stimulations. Images of the index and little finger abduction conditions presented the finger movement at the end point and created an appearance of movement. Similar to previous studies (e.g. ref. 32), the baseline condition consisted of the hand receding and was included to test for any difference in non-specific TMS effect between the two genetic groups. Participants underwent 6 blocks of 27 trials (9 trials of each condition) plus 3 catch trials for a total of 54 trials by condition. Stimuli covered ~19^o^ X 14^o^ degrees of visual angle (Fig. 1).

After the first action observation session, participants performed a procedure previously used to study associative learning^6^,^32^ and that has been shown to change the sensitivity to action observation. In this task, participants were seated in a dimly lit room with their right hand positioned across their body and over a computer keyboard so that their forearm was parallel to the screen to avoid spatial compatibility^41^. Participants were instructed to observe images either displaying a little finger movement or an index finger movement. These images were presented using an identical sequence as the one used in the action observation procedure. Participants were asked to abduct their index finger as quickly as possible when a little finger movement was presented on the computer screen and to execute a little finger abduction when index finger abduction was presented. This training, pairing an observed action with an incompatible executed action, has been shown to create new associations by changing the muscle-specific response observed before training. For instance, pairing the execution of index finger abduction with the observation of little finger abduction should create a new visuomotor association so that observing the little finger would generate motor facilitation in the muscle representation controlling the index finger after training. Therefore, such visuomotor associative learning should change the sensitivity to action observation. Participants completed 6 blocks of 32 trials (16 trials of each observation conditions randomly presented). In each condition, participants had to reach with the appropriate finger and press a key on a modified computer keyboard (see Fig. 1). The distance between the keys was adjusted as a function of the capacity of the participant to effortlessly reach the keys. The key press on each trial recorded the response times.

### MEP preprocessing

Examiners blind to genotypes visually inspected offline signals to remove trials presenting muscle activity prior to the stimulation using homemade functions of the Spike 2 software. Trials presenting muscle activity 500 ms prior to the stimulation were rejected for both muscles. Root mean squares (RMS) were extracted in a window from 0 to 100 ms prior to stimulation. Within blocks, MEPs > 3 SD were removed from further analysis. On average, 6.7% of all trials were rejected within blocks due to outliers or movements artefacts. MEPS were pooled across stimulation intervals in all analyses as no difference was observed between stimulations at 320 and 640 ms in any movement observation condition.

### Data analysis

From a signal detection theory perspective, in order for motor facilitation to be considered muscle-specific it should be sensitive enough to distinguish, solely on the basis of its activity, between two action observation conditions. In fact, a good tuning of the system would maximize the sensitivity of motor representations to discriminate between the observation of a compatible action (signal) and an incompatible action (noise). In order to measure the sensitivity of the FDI and ADM representations, we opted for the sensitivity index (d-prime)^5^, the most common measure of sensitivity in signal detection theory. The d-prime indicates how sensitive a measure is for the discrimination of two experimental conditions, typically a target condition (signal) and a control condition (noise). In the context of action observation, an observed action corresponding to the muscle of interest is considered as the signal while the non-corresponding observed action is considered as the noise. For example, the FDI d-prime is calculated as:


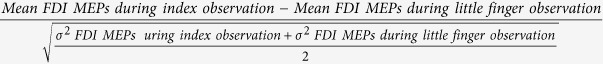


This measure is conceptually similar to measures used by others such as the MEP preference ratio^6^,^32^,^40^ but presents the advantages of being informative about the sensitivity of MEPs and of correcting for individual variance in the data. In fact, to reflect sensitivity, mean proportions must consider the variance of both means in the ratio, as distant means with large standard deviations do not necessarily indicate a greater sensitivity than closer means with small standard deviations. The d-prime considers this factor and indicates accurately how MEPs can be used to discriminate between both action observation conditions. Note that a greater value on this measure indicates a greater difference between the two action observation conditions, thus a greater sensitivity. D-Primes were computed block-by-block to control for any variation in coil position between blocks.

To study the effect of the *BDNF* Val66Met on associative learning, we first assessed the presence and stability of learning without considering genetic groups. This was achieved by studying response time changes during the associative learning task as well as changes in sensitivity (i.e., d-primes) following the associative learning session. To assess the stability of the changes in sensitivity following the learning session, post-training blocks were separated in halves (3 first blocks vs. 3 last blocks) and considered separately. As described previously, visuomotor associative learning should change the sensitivity of the system to discriminate between a compatible observed action and an incompatible observed action. This would be indicated by a decrease in d-primes after the training session, as the two action observation conditions would be rendered similar, hence less discriminable. Furthermore, even in the context of weak pre-training sensitivity (low d-primes), visuomotor associative training should lead to decreased d-primes. In this situation, the development of a new sensitivity to incompatible actions would be evidenced by negative d-primes if the motor representation develops sensitivity to the incompatible conditions after training. Accordingly, negative d-primes would represent the build up of a greater sensitivity to the incompatible condition in comparison to the compatible condition.

Multiple comparisons were carried using the Bonferroni correction. Square root transformation of the d-prime distributions was used so the data would respect the normality postulate.

## Additional Information

**How to cite this article**: Taschereau-Dumouchel, V. *et al*. BDNF Val^66^Met Polymorphism Influences Visuomotor Associative Learning and the Sensitivity to Action Observation. *Sci. Rep*. **6**, 34907; doi: 10.1038/srep34907 (2016).

## Supplementary Material

Supplementary Information

## Figures and Tables

**Table 1:** Demographics of participants.

	Val/Val	Val/Met	Met/Met	p
Female	8	5	3	
Male	8	5	3	
Age (years)	24.5 (1.18)	24.1 (0.92)	24.3 (2.11)	0.97
Education (years)	17.19 (0.54)	15.6 (0.52)	16.0 (1.02)	0.15

Mean (± S.E.M.). P values are the results of univariate analysis of variance (ANOVA).
