# Supplementary material for: BDNF Val66Met Polymorphism Influences Visuomotor Associative Learning and the Sensitivity to Action Observation
Source: Sci Rep. 2016 Oct 5;6:34907. doi: 10.1038/srep34907 (PMC5050503; doi:10.1038/srep34907)
Supplement: Supplementary Information [file srep34907-s1.pdf]

Supplementary information

BDNF Val^{66}Met Polymorphism influences Visuomotor Associative Learning and the Sensitivity to Action Observation

Vincent Taschereau-Dumouchel [1,2,3*], Sébastien Hétu [4], Pierre-Emmanuel Michon [2,3], Etienne Vachon-Presseau [5], Elsa Massicotte [1,2,3], Louis De Beaumont [6,7], Shirley Fecteau [2,3,8,9], Judes Poirier [10,11], Catherine Mercier [2,8], Yvon C. Chagnon [3,12] & Philip L. Jackson [1,2,3]

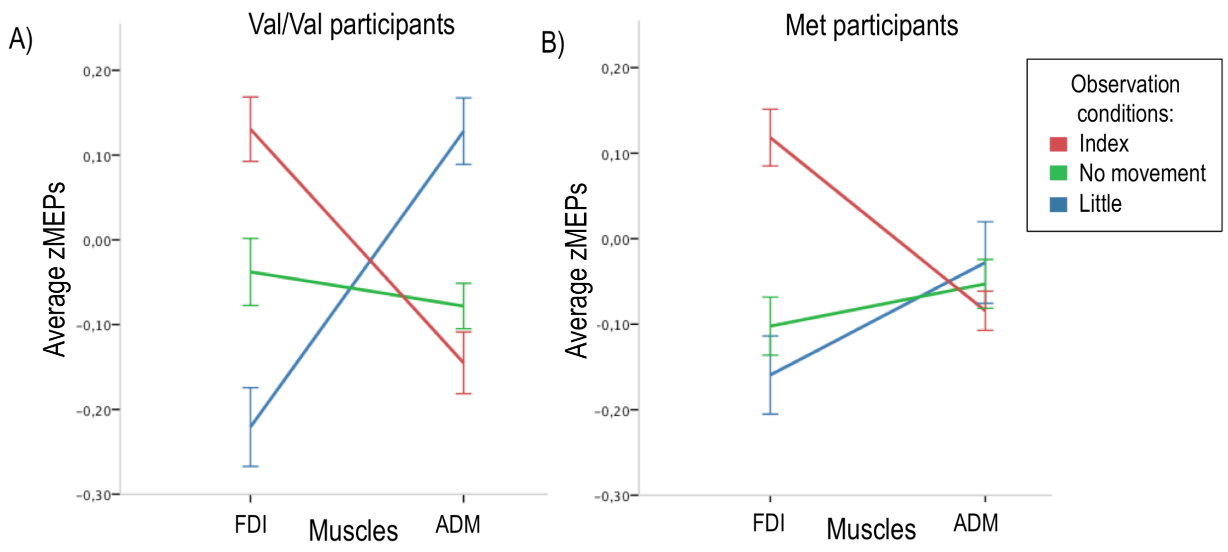

Figure S1. Val66Met polymorphism influences motor-evoked potentials during action observation. (A) Significant motor facilitation in Val/Val participants during the observation of compatible actions in both muscles in comparison to incompatible conditions (ADM: $P = .001$; FDI: $P = .0003$) or the no movement condition (ADM: = .003; FDI: $P = .01$). (B) In Met participants, significant motor facilitation was only observed in the FDI muscle (Compatible vs. Incompatible action: $P = .0005$; Compatible vs. No movement condition: $P = .001$), but not in the ADM muscle (Compatible vs. Incompatible action: $P = .20$; Compatible vs. No movement condition: $P = 0.53$). Error bars indicate standard errors of the mean. While group averaged MEPs is probably the metric the most commonly used in the MNS literature, this measure offers minimal information regarding the within-participant sensitivity to action observation (e.g., within-subject difference between a corresponding action and non-corresponding action). Therefore, the sensitivity index (d-prime) was preferred to document individual differences (see Data analysis).

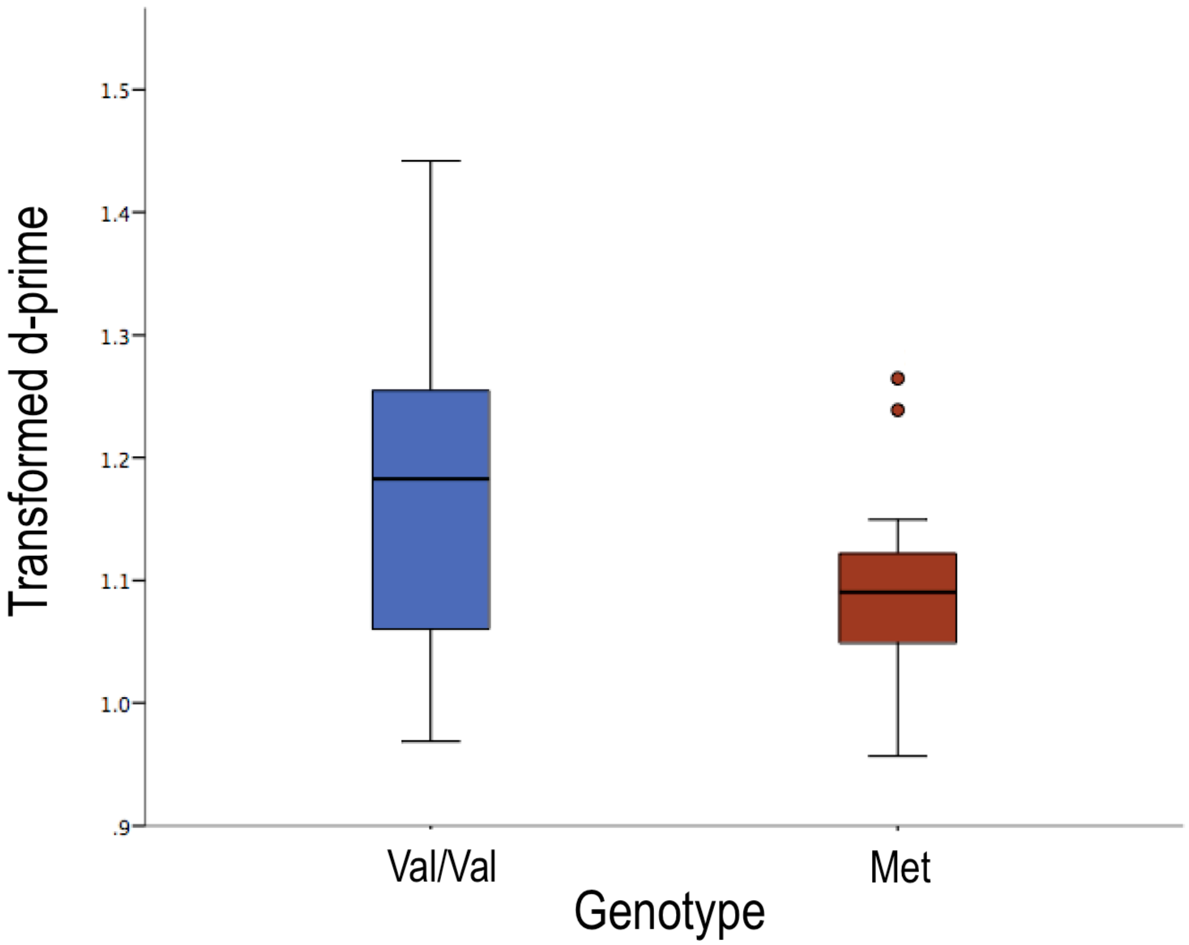

Figure S2. Boxplot depicting the transformed d-prime before training as a function of the *BDNF* Val66Met genotype.

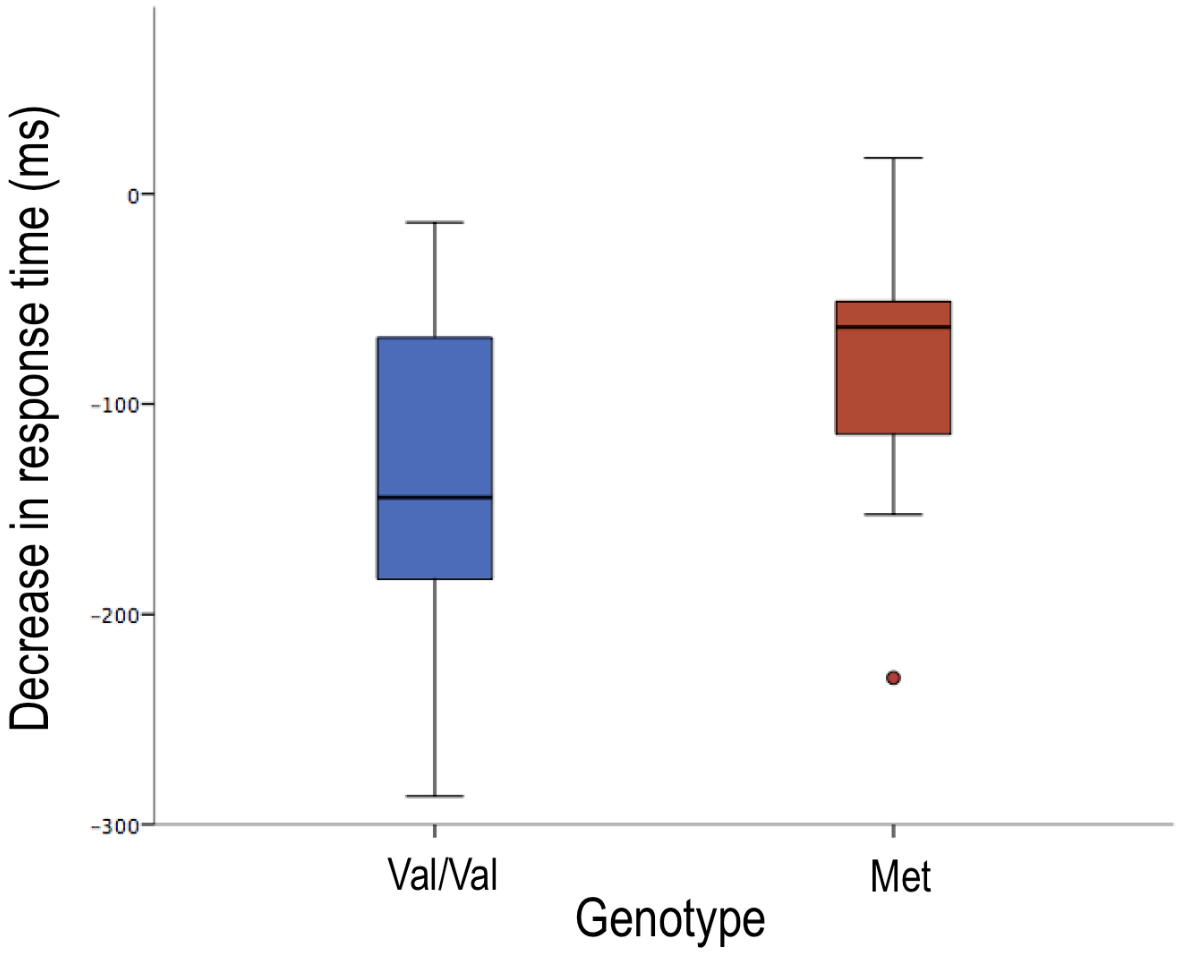

Figure S3. Boxplot depicting the decrease in response time as a function of the *BDNF* Val66Met genotype.
